# The predictive value of centre tumour CD8^+^ T cells in patients with hepatocellular carcinoma: comparison with Immunoscore

**DOI:** 10.18632/oncotarget.5801

**Published:** 2015-09-22

**Authors:** Cheng Sun, Jing Xu, Jiaxi Song, ChaoQun Liu, Jinyu Wang, Chenchun Weng, Haoyu Sun, Haiming Wei, Weihua Xiao, Rui Sun, Zhigang Tian

**Affiliations:** ^1^ Institute of Immunology and The CAS Key Laboratory of Innate Immunity and Chronic Disease, School of Life Sciences and Medical Center, University of Science & Technology of China, Hefei, Anhui, China; ^2^ Collaborative Innovation Center for Diagnosis and Treatment of Infectious Diseases, State Key Laboratory for Diagnosis and Treatment of Infectious Diseases, First Affiliated Hospital, College of Medicine, Zhejiang University, Hangzhou, Zhejiang, China; ^3^ Sun Yat-Sen University Cancer Center, State Key Laboratory of Oncology in South China, Guangzhou, China

**Keywords:** CD8^+^ T cells, Immunoscore, HCC, prognostic indicator, TNM

## Abstract

The increasing evidences suggest that Immunoscore(IS), a combinatorial density analysis of CD8^+^ and CD3^+^ cells in the centre and invasive margin of tumour (CT and IM), has an advantage over the currently used tumour staging methods in a variety of tumours; however, IS in hepatocellular carcinoma remains unreported. In this study, IS was performed on serial sections from two HCC cohorts (total 449) and compared with current tumour staging systems. Kaplan–Meier curves illustrate a positive association between a higher IS (IS≥2) and longer survival of HCC patients. Although the IS was highly related to the outcome of patients, however, IS seems not to be the optimal prognostic factor when compared with the CD8_CT_. As noted, among CD8_CT_, CD8_IM_, CD3_CT_, CD3_IM_ and IS, CD8_CT,_ as an independent indicator, demonstrated the highest prognostic impact on both DFS and OS in our Cox multivariate regression analysis (*P*< 0.0001). In our study, the minimum cut-off value was 93 CD8_CT_ cells per mm^2^, to be used to divide the patients into CD8_CT_^Hi^ group and CD8_CT_^Lo^ group in clinical settings. Our results suggest that CD8_CT_ densities analysis notably improved the accuracy of survival prediction with convenience of clinical manipulation in HCC.

## INTRODUCTION

Liver cancer is a leading cause of cancer deaths, accounting for more than 700,000 deaths per year worldwide. Current clinical risk predictions in hepatocellular carcinoma (HCC) are based on histopathological evaluation of the primary tumour tissue obtained during surgery. The Tumour-Node-Metastasis (TNM) staging system of the American Joint Committee on Cancer (AJCC)/International Union Against Cancer (UICC) has been verified to be valuable in the outcome estimation of patients with a variety of tumours. However, this traditional cancer staging system, which is based only on tumour invasion parameters, provides limited information in the estimation of the post-operative outcome of HCC patients. In some tumours, patients within the same histological tumour stage had clinical outcomes that varied significantly [1-2]. The focal point of this traditional classification is primarily on the tumour cells and pays essentially no attention to the effects of the host immune response. Recently, considerable data from large cohorts of various tumours have demonstrated that the number, type and location of tumour-infiltrating lymphocytes (TILs) are essential for the prediction of clinical outcome [3-8]. Based on these studies, Galon et al. recommended that IS should be used as a new prognostic tool and a component of cancer classification [5]; IS was further confirmed to be beneficial for the prognosis of disease-free survival (DFS) and overall survival (OS), particularly in early-stage cancers [9]. The fundamental parameter of the IS classification includes the density of the CD3^+^ and CD8^+^ T cells at the centre of the tumour (CT) and at its invasive margin (IM), which has previously been extensively used in a variety of tumour types, including colon, rectal, melanoma and breast cancers [3-5, 7-10].

However, the predictive role of IS in patients with HCC who underwent resection remains unknown. In this study, IS was highly correlated to the outcome of HCC patients (*n* = 359) if compared with TNM or BLCL staging, however, IS seems not to be the best prognostic factor. Among CD8_CT_, CD8_IM_, CD3_CT_, CD3_IM_ and IS, CD8_CT_ works as an independent indicator, demonstrating the highest prognostic impact not only on DFS and OS, but also on tumour relapse, tumour size and serum levels of ALT and AST in Cox multivariate regression analysis. We recommend that optimum cut-off value is 93 CD8_CT_ cells per mm^2^, to be used to divide the patients into CD8_CT_^Hi^ group and CD8_CT_^Lo^ group in clinical settings. Our results suggest that CD8_CT_ densities analysis improved the survival prediction with convenience of clinical manipulation in HCC.

## RESULTS

### Positive correlations of the densities of CD3^+^ or CD8^+^ cells/mm^2^ in centre tumour but not peritumour to overall survival in cohort 1

**Figure 1:**
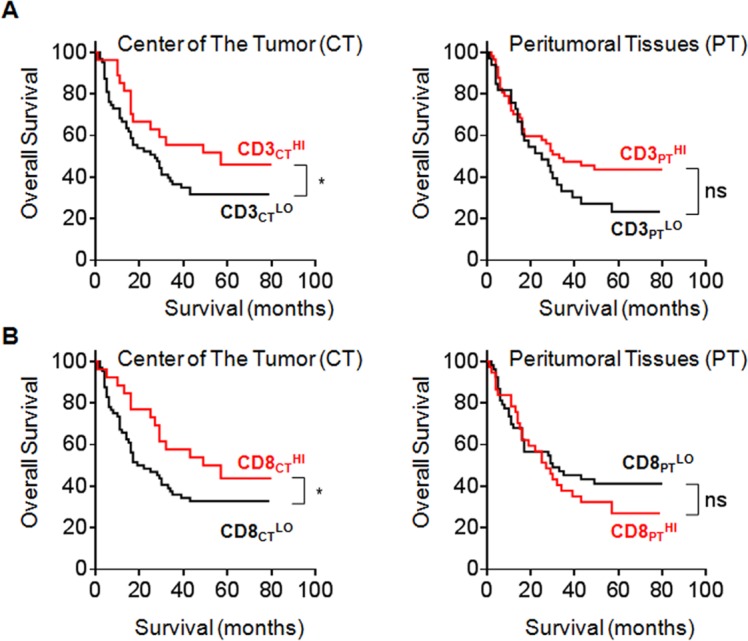
Correlation between the number of CD3^+^ or CD8^+^ cells/mm^2^ and OS in CT and PT regions **A.**, **B.** Kaplan-Meier curves for the duration in months of OS according to CD3^+^ **A.** and CD8^+^ **B.** cells densities evaluated in each tumour regions (CT and PT) (High densities, red line; Low densities, black line). Cut-off values were 214, 375, 97, and 186 cells/mm^2^ for CD3_CT_, CD3_PT_, CD8_CT_ and CD8_PT_, respectively. In **A.** and **B.**, log-rank statistical test, *, *P* < 0.05.

It has been observed that CD8^+^ lymphocytes infiltrated in HCC, which likewise has been correlated with the tumour progression [11]. However, the different densities of immune cells within different tumour regions and the clinical outcome of patients have never been reported in HCC. In this study, we used commercially available tissue microarray (TMA) from one HCC cohort with 90 patients and examined the distributions of CD3^+^ T cells and CD8^+^ T cells in HCC tissues through two identical TMAs, followed by immunostaining. The CD3^+^ and CD8^+^ T cells in the centre tumour (CT) and peritumour tissues (PT) from 12 representative samples are shown in Figure S1A-B, which demonstrated that the distribution and density of these lymphocytes varied in different HCC areas. Cox regression with time-to-event outcome analysis demonstrated that no positive correlations of the number of CD3^+^ or CD8^+^ cells/mm^2^ in the CT and PT regions to overall survival (OS) have been identified primarily due to the small sample size (Table. S2). To further assess the predictive potential of CD3^+^ or CD8^+^ cell densities in different tumour regions (CT and PT), the patients were divided into two groups using the minimum *P*-value cut-off values for CD3 or CD8 densities in each tumour region (Cut-off values were 214, 375, 97, and 186 for CD3_CT_, CD3_PT_, CD8_CT_ and CD8_PT_, respectively). As expected, the survival curve by sub grouping showed that higher CD3^+^ or CD8^+^ cell densities in the CT (Figure 1A) but not PT (Figure 1B) regions from the HCC patients were correlated with longer OS (log-rank test corrected *P* < 0.05 for both CD3_CT_ and CD8_CT_). Taken together, this small cohort study suggests that the distribution and their densities of CD3^+^ and CD8^+^ T cells in centre tumour regions have the predictive value for HCC progression.

### CD3^+^ or CD8^+^ T cells in invasive margin and centre tumour in a large cohort 2

**Figure 2:**
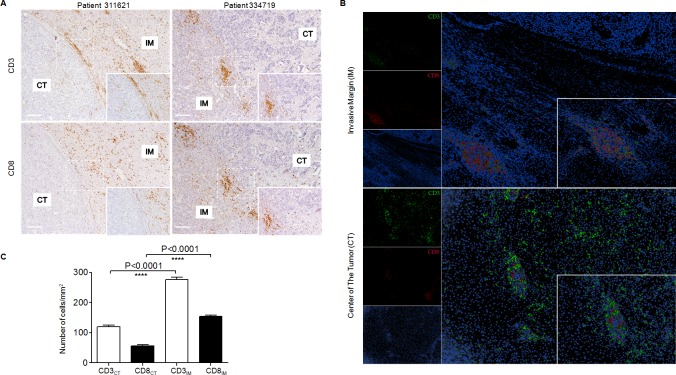
Different densities and locations of CD3^+^ or CD8^+^ cells in HCCpatients **A.** Adjacent sections of paraffin-embedded hepatoma samples stained with anti-CD3 or anti-CD8 antibodies. Representative micrographs showing infiltrating CD3^+^ lymphocytes (Top) andCD8^+^ lymphocytes (Bottom) in the IM regions from 2 HCC patients (Left Pts311621: Capsule positive; Right Pts334719: Capsule negative). Original magnifications: ×10, ×20. **B.** Representative micrographs showing double staining of CD3^+^ and CD8^+^ cells in the IM region (Top) and CT region (Bottom), and Adobe Photoshop was used to convert the immunohistochemical staining to fluorescence. Original magnifications: ×10, ×40. **C.** Density of CD3^+^ (White) and CD8+ (Black) cells in the IM and CT regions were shown, respectively. Statistical significance were analysed by the one-way ANOVA test. ****, *P* < 0.0001.

It has been recognised that immune cells are scattered in the invasive margin (IM) tumour in addition to the CT and PT regions; however, the predictive role of CD3^+^ or CD8^+^ T cell density in the IM regions in HCC has not been addressed. Since the commercial supply of the HCC sample in Cohort 1 contains only PT and CT but not IM regions, we further investigated the role of the IM-infiltrating CD3^+^ or CD8^+^ T cells using TMAs containing CT and IM regions resection specimens from 359 HCC patients (Cohort 2). The strategy of designating tissue cores for TMAs from the tumour centre and the invasive margin was shown in Figure S3A. These tissue sections may clearly be divided into CT and IM regions and clearly demonstrate high or low densities of CD3^+^ or CD8^+^ T cells, according to the method described by Galonet et al. [4]. For the purpose of accuracy, in addition to the TMAs, the whole sections from 21 HCC patients were also used to investigate the expression of CD3^+^ and CD8^+^lymphocytes. Two representative sections exhibited the distinct high-density infiltrating areas of immune cells in the IM regions (Figure 2A). The double staining of CD3^+^ and CD8^+^ cells in the HCC tissue demonstrated that the lymphocyte densities differed significantly between the CT and IM regions, with a higher density and larger area of lymphocytes in the IM region (Figure 2B, Figure S3B). Consistent with this finding, the average number of CD3_IM_ or CD8_IM_ T cells was significantly higher than those of CD3_CT_ or CD8_CT_ T cells (*P* < 0.0001 for both) (Figure 2C); together, these findings suggest the IM T cell is a potential candidate indicator in the prediction of HCC prognosis.

**Figure 3:**
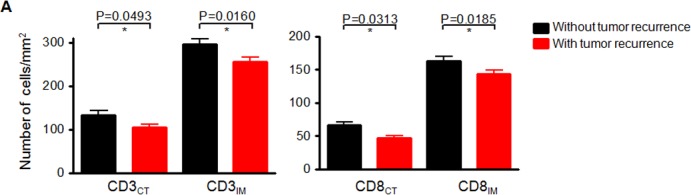
Negative correlation between the number of CD3^+^ or CD8^+^ cells/mm^2^ and tumour recurrence in HCC patients **A.** Comparison of the immune cell densities in the CT and IM regions from patients with (Red bars) or without (Black bars) tumour recurrence. Comparisons were performed using Mann-Whitney test, *P* values are shown.

To further assess the impact of different density of immune cells in each tumour region in HCC patients, we analysed the correlation between the immune cell densities in the CT and IM regions and the survival time of the patients from Cohort 2. As shown in TableS2, statistical analysis demonstrated that significant positive correlations of the number of CD3^+^ or CD8^+^ cells/mm^2^ in both CT and IM regions to disease-free survival (DFS) or overall survival (OS) have been identified (*P* < 0.0001 in all comparisons) due to the relatively large sample size. Moreover, the patients with higher density of CD3^+^ or CD8^+^cellin both CT and the IM regions had significantly lower tumour recurrence (*n* = 184), and those with lower density of T cells had higher tumour recurrence (*n* = 175) (Figure 3A).

**Figure 4:**
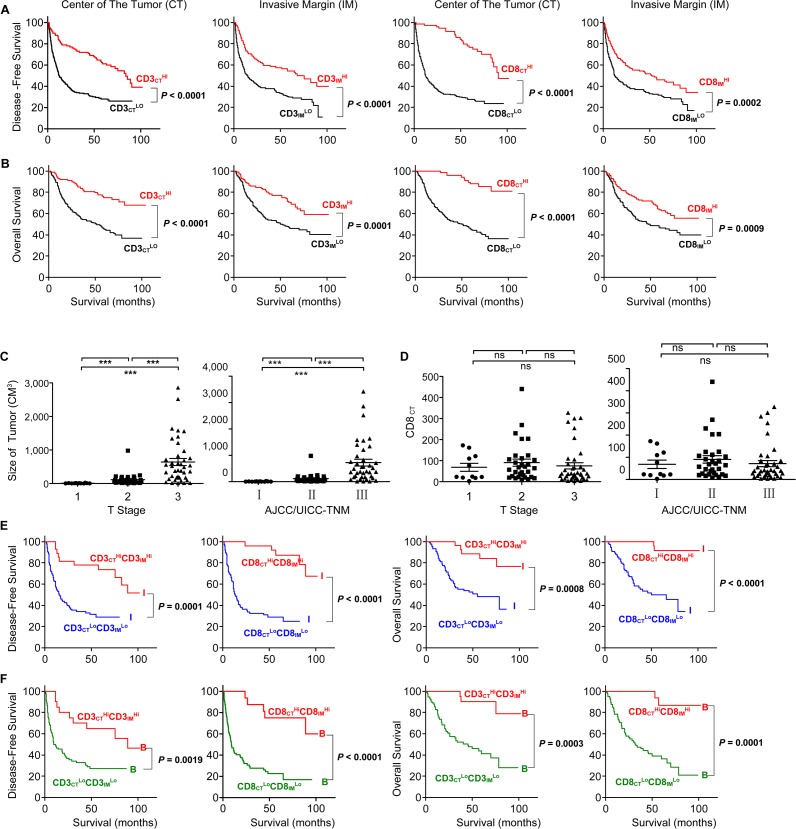
Increased survival time for the patients with high densities of CD3^+^ or CD8^+^ cells Kaplan-Meier curves for the duration in months of DFS **A.** and OS **B.** according to CD3^+^ or CD8^+^ cells densities evaluated in each tumour regions (CT and IM) (High densities, Red line; Low densities, Black line). The tumour size **C.** and the density of CD8^+^ lymphocytes **D.** in the CT regions were compared with different T stages and AJCC/UICC-TNM stages, respectively. **E.** The TNM-I stage HCC patients were divided into two groups according to different densities of CD3^+^ or CD8^+^ cells in the combined tumour regions (CT and IM) (e.g. CD3_CT_^Hi^CD3_IM_^Hi^, CD3_CT_^Lo^CD3_IM_^Lo^, CD8_CT_^Hi^CD3_IM_^Hi^ and CD8_CT_^Lo^CD3_IM_^Lo^; LoLo, blue lines; HiHi, red lines). Kaplan-Meier curves illustrated the durations of OS and DFS in each group. **F.** The BLCL-B stage HCC patients were divided into two groups according to different densities of CD3^+^ or CD8^+^ cells in the combined tumour regions (CT and IM) (e.g. CD3_CT_^Hi^CD3_IM_^Hi^, CD3_CT_^Lo^CD3_IM_^Lo^, CD8_CT_^Hi^CD3_IM_^Hi^ and CD8_CT_^Lo^CD3_IM_^Lo^; LoLo, green lines; HiHi, red lines). Kaplan-Meier curves illustrated the durations of OS and DFS in each group. In Kaplan-Meier curves, log-rank statistical test, *P* values are shown.

We next divided these patients into two groups according to the minimum *P*-value cut-offs for CD3^+^ or CD8^+^ density (164, 324, 93, and 164 for CD3_CT_, CD3_IM_, CD8_CT_, CD8_IM_, respectively) in each tumour region. The patients with high densities of CD3^+^or CD8^+^T cells in both CT and IM regions in Cohort 2 exhibited significantly longer DFS and OS (*P* < 0.0001 for all comparisons) (Figure 4A, 4B), almost similar to the performance of CD3^+^or CD8^+^ T cells in CT region in Cohort 1 (Figure 1). Importantly, among the four positive indicators (CD8_CT_^Hi^, CD8_IM_^Hi^, CD3_CT_^Hi^, CD3_IM_^Hi^), CD8_CT_^Hi^ and CD3_CT_^Hi^ were more efficient to predict DFS and OS (all log-rank test corrected *P* < 0.0001) when compared with CD8_IM_^Hi^ and CD3_IM_^Hi^ on Kaplan-Meier curves.

Univariate analysis further showed that TNM staging, T stage, tumour number, thrombus, CD8_CT_^Hi^, CD8_IM_^Hi^, CD3_CT_^Hi^, and CD3_IM_^Hi^ significantly influenced DFS and OS (*P* < 0.05 for all comparisons in Table 1), respectively. However, among these indicators, lymphocytes in CT region were more positively influential on DFS and OS than those in IM region, and CD8^+^ T cells had more impact on DFS and OS than CD3^+^ T cells (HRs were 0.5 and 0.49 for CD3_IM_, 0.6 and 0.58 for CD8_IM_, 0.38 and 0.35 for CD3_CT_, 0.23 and 0.16 for CD8_CT_ for DFS and OS, respectively; all *P* < 0.0004; Table 1). Next, patients with high density of CD3 or CD8 in both CT and IM regions were classified as “HiHi” group; patients with low density of each marker in both regions were classified as “LoLo” group; patients with a high density of one marker in either one region (CT or IM) were classified as “Het” group. It was also noted that HiHi for CD3^+^ T cells (HRs = 0.24) and HiHi for CD8^+^ T cells (HRs = 0.17) had more impact on DFS than LoLo groups (*P* < 0.0001 for all comparisons; Table 1).

**Table 1:** Univariate Analysis of DFS and OS Among Patients With Liver Cancer (cohort 2) According to Clinical or Immune Parameters

		DFS			OS		
Parameter	No. of pts (%)	HR	95% CI	P*	HR	95% CI	P*
**Clinical parameters**							
Gender (Male)	318(88.6)	0.9	0.6 to 1.36	0.6134	0.75	0.48 to 1.18	0.2141
Age (y)		0.93	0.81 to 1.07	0.3328	0.95	0.8 to 1.12	0.5162
<50	175(48.9)	1.0	(reference)	0.1996	1.0	(reference)	0.5675
50-60	99(27.7)	1.11	0.82 to 1.52	0.5041	1.15	0.8 to 1.65	0.4541
60-70	66(18.4)	1.05	0.74 to 1.48	0.7975	0.96	0.62 to 1.47	0.8492
70	18(11.4)	0.5	0.24 to 1.02	0.0574	0.67	0.31 to 1.46	0.3164
Number (multiple/single)	90(25.5)	2.28	1.72 to 3.03	<0.0001†	2.31	1.66 to 3.21	<0.0001†
Metastasis (Y/N)	34(10.4)	1.62	1.06 to 2.48	0.0264†	1.49	0.89 to 2.51	0.1309
Tumour Thrombus (Y/N)	43(12.3)	2.94	2.03 to 4.26	<0.0001†	4.44	2.96 to 6.67	<0.0001†
Diameter of Tumor≤5cm	144(40.8)	1.0	(reference)		1.0	(reference)	
Diameter of Tumor >5cm	209(59.2)	1.43	1.1 to 1.86	0.0082†	1.69	1.23 to 2.31	0.0013†
Pathology Grading		1.06	0.83 to 1.35	0.6267	1.26	0.95 to 1.68	0.1079
1	17(4.9)	1.0	(reference)	0.3076	1.0	(reference)	0.3141
1.5	7(2.0)	3.97	1.26 to 12.53	0.0190†	7.73	1.29 to 46.39	0.0253†
2	174(50.6)	1.54	0.72 to 3.32	0.2653	4.19	1.03 to 17.04	0.0456†
2.5	45(13.1)	1.38	0.6 to 3.17	0.4475	3.44	0.8 to 14.9	0.0984
3	94(27.3)	1.71	0.78 to 3.75	0.1775	4.92	1.19 to 20.3	0.0275†
3.5	4(1.2)	1.31	0.27 to 6.31	0.7365	4.82	0.68 to 34.25	0.1157
4	3(0.9)	0.79	0.1 to 6.43	0.8266	3.41	0.31 to 37.59	0.3174
HBV (Y/N)	320(91.2)	1.16	0.72 to 1.88	0.5506	0.76	0.46 to 1.26	0.2832
HCV (Y/N)	7(2.0)	1.38	0.57 to 3.36	0.4763	0.43	0.06 to 3.04	0.3938
AFP <100ng/ml	159(55.8)	1.0	(reference)		1.0	(reference)	
AFP ≥100ng/ml	126(44.2)	1.17	0.87 to 1.58	0.3059	1.43	1.00 to 2.04	0.0478
UICC (TNM) stage		1.28	1.14 to 1.44	<0.0001†	1.33	1.16 to 1.53	<0.0001†
0-I	182(50.1)	1.0	(reference)	<0.0001†	1.0	(reference)	<0.0001†
II	27(7.4)	2.2	1.4 to 3.47	0.0007†	2.13	1.23 to 3.69	0.0067†
III	110(30.3)	1.81	1.34 to 2.45	0.0001†	2.18	1.54 to 3.1	<0.0001†
IV	44(12.1)	1.89	1.2 to 2.97	0.0060†	1.8	1.03 to 3.16	0.0399†
BCLC stage		1.1	0.92 to 1.31	0.2979	1.15	0.93 to 1.43	0.1874
A	103(28.8)	1.0	(reference)	0.5170	1.0	(reference)	0.3075
B	164(45.8)	1.18	0.86 to 1.62	0.3144	1.33	0.9 to 1.96	0.1536
C	91(25.4)	1.21	0.84 to 1.73	0.3044	1.34	0.86 to 2.08	0.1925
**Immune parameters**							
Immune score		1.63	1.44 to 1.85	<0.0001†	1.69	1.45 to 1.96	<0.0001†
0	143(39.8)	11.05	4.44 to 27.48	<0.0001†	29.17	4.05 to 210.15	0.0008†
1	68(18.9)	5.8	2.27 to 14.8	0.0002†	20.66	2.83 to 151.01	0.0028†
2	95(26.5)	4.26	1.7 to 10.69	0.0020†	11.36	1.56 to 82.97	0.0166†
3	29(8.1)	3.3	1.2 to 9.1	0.0213†	7.63	0.95 to 61.04	0.0554
4	24(6.7)	1.0	(reference)	<0.0001†	1.0	(reference)	<0.0001†
Immune score							
≥2	148(41.1)	1.0	(reference)		1.0	(reference)	
<2	211(58.6)	2.56	1.93 to 3.41	<0.0001	2.99	2.1 to 4.26	<0.0001
CD3_CT/IM_							
(LoLo)	191(53.2)	1.0	(reference)		1.0	(reference)	
(Het)	123(34.3)	0.46	0.34 to 0.62	<0.0001†	0.5	0.35 to 0.7	<0.0001†
(HiHi)	45(12.5)	0.23	0.14 to 0.39	<0.0001†	0.18	0.09 to 0.38	<0.0001†
CD8_CT/IM_							
(LoLo)	171(47.6)	1.0	(reference)		1.0	(reference)	
(Het)	152(42.3)	0.46	0.35 to 0.61	<0.0001†	0.46	0.33 to 0.64	<0.0001†
(HiHi)	36(10)	0.16	0.09 to 0.31	<0.0001†	0.1	0.04 to 0.27	<0.0001†
CD3_IM_							
(Low)	246(68.5)	1.0	(reference)		1.0	(reference)	
(High)	113(31.5)	0.5	0.37 to 0.67	<0.0001†	0.49	0.34 to 0.71	0.0002†
CD8_IM_							
(Low)	206(47.4)	1.0	(reference)		1.0	(reference)	
(High)	153(42.6)	0.6	0.46 to 0.79	0.0003†	0.58	0.42 to 0.81	0.0011†
CD3_CT_							
(Low)	259(72.4)	1.0	(reference)		1.0	(reference)	
(High)	100(27.6)	0.38	0.27 to 0.53	<0.0001†	0.35	0.23 to 0.53	<0.0001†
CD8_CT_							
(Low)	288(80.2)	1.0	(reference)		1.0	(reference)	
(High)	71(19.8)	0.23	0.15 to 0.36	<0.0001†	0.16	0.08 to 0.31	<0.0001†

NOTE: All categorical covariates were transformed into numeric codes before they were entered into the Cox model. Numeric codes are as follows: Gender: female = 0, male = 1. Age: less than 50 = 1, 50 ∼ 60 = 2, 60 ∼70 = 3, more than 70 = 4. Number: single = 1, multiple = 2. Metastasis: N = 1, Y = 2. Tumour thrombus: N = 0, Y = 1. Diameter of Tumor: less than 5cm = 1, more than 5cm = 2. HBV: N = 0, Y = 1. HCV: N = 0, Y = 1. AFP: less than 100ng/ml = 1, more than 100ng/ml = 2. UICC-TNM stage: stage I = 1, stage II = 2, stage III = 3, stage IV = 4. BCLC stage: stage A = 1, stage B = 2, stage C = 3. Immune score: CD3_CT/IM_ CD8_CT/IM_ (CT, center of tumor; IM, invasive margin): Im2, 3, 4 = 1, Im0, 1 = 2. CD3_CT/IM_: low = 1, het = 2, high = 3. CD8_CT/IM_: low = 1, het = 2, high = 3. CD3_IM_: low = 1, high = 2. CD8_IM_: low = 1, high = 2. CD3_CT_: low = 1, high = 2. CD8_CT_: low = 1, high = 2.

^a^For patients with data available on TMA analyses.

Abbreviations: DFS, disease-free survival; OS, overall survival; HR, hazard ration; CI, confidence interval; UICC-TNM, International Union Against Cancer–TNM (staging systems);

† Significant.

One thing to note is that Univariate analysis showed that HRs did not progressively correlate to the severity of TNM stage (in response to the increasing staging from I to IV, the associated HRs were 1, 2.2, 1.81, 1.89 for DFS, and 1, 2.13, 2.18, 1.8 for OS, respectively) (Table 1), indicating that TNM classification might not be the accurate staging system in predicting the outcome of HCC patients. On the other hand, although there was a good correlation of the tumour size with T stages or UICC-TNM stages (Figure 4C), the CD8_CT_ number alone was not correlated with these stages (Figure 4D), suggestive of TNM staging system could not indicated the immune contexture in the centre of tumour.

### CD8_CT_ is a better indicator than IS to predict the prognosis of HCC

**Figure 5:**
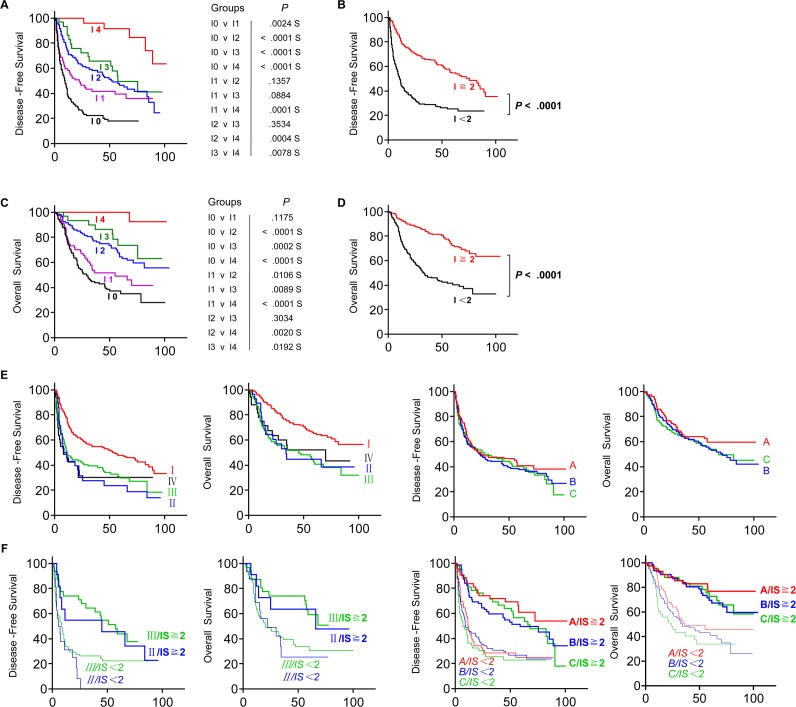
Increased survival time for the patient with high Immunoscore (IS) **A.** DFS and **C.** OS of patients with IS 0 (Black), IS 1 (Pink), IS 2 (Blue), IS 3 (Green) or IS 4 (Red) were shown. **B.** DFS and **D.** OS of patients with IS ≥2 (Red) and IS < 2 (Black). (**E.**, two lefts) DFS and OS of patients with different TNM stages (TNM-I, Red; TNM-II, Blue; TNM-III, Green; TNM-IV, Black), and (**E.**, two rights) DFS and OS of patients with BLCL-A (Red), BLCL-B (Blue) or BLCL-C (Green) stages. **F.** DFS and OS of TNM-II (Blue) or -III (Green), or BLCL-A (Red), -B (Blue) or -C (Green) HCC patients with IS ≥2 (Red) and IS < 2 (Black) were illustrated by Kaplan-Meier curves.

As expected, a combinational analysis of CD3 (e.g. CD3_CT_^Hi^ CD3_IM_^Hi^ vs. CD3_CT_^Lo^ CD3_IM_^Lo^) efficiently predicted the HCC survival even within same TNM-I stage (Figure 4E) or BLCL-B stage (Figure 4F) patients, which might be repeated by a combinational analysis of location and density of CD8^+^ T cells [7-8]. We then further investigated whether the combined analysis (e.g. Immunoscore (IS)) of both high and low CD3^+^ and CD8^+^ T cell densities in tumour regions (CT or IM) could improve the prediction of patient outcome, which has been successfully used to predict outcome of colon cancer and rectal cancer [5, 10, 12]. The patients were divided into the HiHi (high densities in both regions) and LoLo (low densities in both regions) groups, which had been used in Figure 4; the highest score was 4 (e.g., CD3^HiHi^ plus CD8^HiHi^), and the lowest score was 0 (e.g., CD3^LoLo^ plus CD8^LoLo^). As shown in Figure 5A, the IS of the 359 HCC sections were divided into IS-0 (*n* = 143), IS-1 (*n* = 68), IS-2 (*n* = 95), IS-3 (*n* = 29) and IS-4 (*n* = 24). We found the DFS (Figure 5A) and OS (Figure 5C) of patients were gradually prolonged if correspondent IS increased: IS-4 (I4) with the longest survival. A strong association was identified between higher IS (IS≧2) and longer DFS and OS, and between lower IS (IS < 2) and shorter DFS and OS (*P* < 0.0001 for all comparisons) (Figure 5B, 5D), looking like IS was a satisfied marker in predicting survival. In our Cohort 2, however, we did not observe any strong differences in survival of patients subgrouping by TNM or BCLC staging (Figure 5E). Interestingly, when we used IS-2 as a cut-off value, the TNM stage II or III of HCC could be divided into two distinct groups: the patients with IS≧2 in TNM I or TNM III clearly exhibited longer survival compared with the patients with IS < 2 (Figure 5F). Consistent with the TNM classification, the patients with IS≧2 in BLCL-A, B or C also clearly exhibited longer survival (Figure 5F). These findings suggest that IS is a prognostic factor superior to or compensate for the AJCC/UICC TNM classification and BLCL staging for the current classifications in HCC, similar to findings in colorectal cancer and rectal cancer [8, 10].

**Figure 6:**
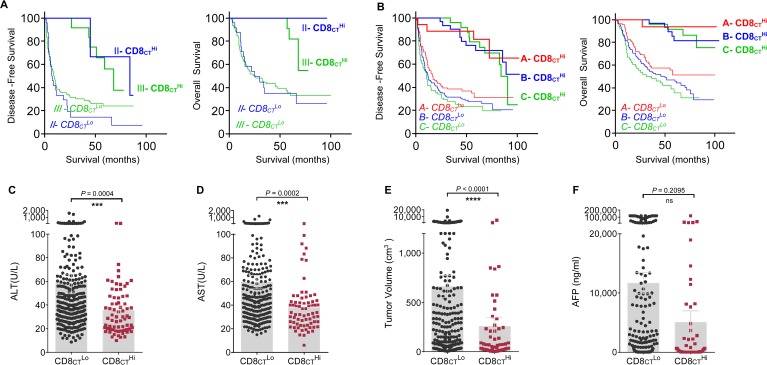
CD8CT directly predicts survival and associates with clinical characteristics of HCC **A.** DFS and OS of TNM-II (Blue) or -III (Green), or **B.** BLCL-A (Red), -B (Blue) or -C (Green) HCC patients with CD8_CT_^HI^ or CD8_CT_^Lo^ were illustrated by Kaplan-Meier curves. The levels of ALT **C.**, AST **D.**, the tumour volume **E.** and AFP **F.** for all HCC patients with CD8_CT_^Lo^ (Black) and CD8_CT_^Hi^ (Red) were analyzed. Cut-off values for CD8_CT_^Lo^ and CD8_CT_^Hi^were 93 cells/mm^2^. Comparisons were made using the Mann Whitney test, *P* values are shown, ***, *P* < 0.001, ****, *P* < 0.0001; ns, no significant.

To further verify whether IS was an independent predictive tool, we built a multivariate analysis model combining CD8_CT_, CD8_IM_, CD3_CT_, CD3_IM_, IS and all clinical pathological variables with P < 0.05 from the Univariate analyses. Interestingly, only CD8_CT_, CD8_IM_ and CD3_IM_ remained significantly correlated to DFS (*P* = < 0.0001, 0.0107, 0.0017, respectively; Table 2) and only CD8_CT_ and CD8_IM_significantly correlated to OS (*P* < 0.0001 and 0.0013, respectively; Table 2) in the model after backward-based Cox multivariate analysis. We then performed Cox multivariate regression analysis by adding CD8_CT_, CD8_IM_, CD3_IM_ and IS into a model. Interestingly, only CD3_IM,_ CD8_IM_ and CD8_CT_ remained significantly associated with DFS (HR: 0.43, 0.71, 0.17, respectively; *P* = 0.0004,0.049, < 0.0001; Table 2). Although IS was highly related to the outcome of HCC patients, however, the IS seemed not to be a best prognostic factor than CD8_IM_ and CD8_CT_. Central CD8^+^ TIL density has independent and the highest significantly prognostic impact on DFS and OS (Both *P* < 0.0001; Table 2). When we nominated CD8_CT_ density as a cut-off value, patients with the same TNM stage II or III could be clearly divided into two distinct groups: the patients with higher CD8_CT_ density in TNM II or III stage clearly exhibited longer survival compared with the patients with lower CD8_CT_ density (Figure 6A). A similar effect of division were also observed in the BLCL-A, B or C stage (Figure 6B), indicating that CD8_CT_ density is a more favourite indicator for predictions.

**Table 2:** Multivariate Cox Proportional Hazard Analysis for DFS and OS Among Patients With Liver Cancer From Cohort 2

	DFS			OS		
Variable^a^	HR	95% CI	P*	HR	95% CI	P*
UICC (TNM) stage	1.14	0.99 to 1.31	0.0706	1.2	1.01 to 1.41	0.0335†
BCLC stage	1.14	0.95 to 1.36	0.1584	1.25	1.01 to 1.55	0.0446†
Immune score	2.42	1.81 to 3.24	<0.0001†	2.82	1.97 to 4.04	<0.0001†
Variable						
CD3_IM_	0.43	0.26 to 0.69	0.0004†			
CD8_CT_	0.17	0.09 to 0.3	<0.0001†	0.16	0.09 to 0.31	<0.0001†
CD8_IM_	0.71	0.5 to 1	0.0490†	0.59	0.42 to 0.82	0.0014†
Immune score	0.64	0.38 to 1.09	0.1029			
Final model Before backward selection						
T stage	1.09	0.97 to 1.23	0.1676	1.17	1.01 to 1.35	0.0313†
N stage	1.57	1 to 2.48	0.0520	1.53	0.88 to 2.65	0.1312
Tumour thrombus	1.98	1.32 to 2.97	0.0010†	3.35	2.14 to 5.25	<0.0001†
CD3_IM_	0.44	0.26 to 0.75	0.0022†	0.66	0.35 to 1.25	0.1979
CD8_IM_	0.58	0.39 to 0.86	0.0069†	0.63	0.39 to 1.01	0.0538
CD3_CT_	0.95	0.59 to 1.55	0.8407	1.18	0.66 to 2.11	0.5725
CD8_CT_	0.14	0.07 to 0.28	<0.0001†	0.14	0.06 to 0.36	<0.0001†
Immune score	0.56	0.3 to 1.04	0.0671	0.87	0.41 to 1.86	0.7183
After backward selection						
T stage				1.16	1.01 to 1.33	0.0369†
N stage	1.51	0.96 to 2.37	0.0742			
Tumour thrombus	2.16	1.46 to 3.19	0.0001†	3.44	2.21 to 5.37	<0.0001†
CD3_IM_	0.45	0.28 to 0.74	0.0017†			
CD8_IM_	0.6	0.4 to 0.89	0.0107†	0.56	0.39 to 0.8	0.0013†
CD8_CT_	0.14	0.08 to 0.27	<0.0001†	0.17	0.09 to 0.34	<0.0001†
Immune score	0.6	0.34 to 1.05	0.0742			

NOTE: All categorical covariates were transformed into numeric codes before they entered into the Cox model. Numeric codes are as follows: UICC-TNM stage: stage I = 1, stage II = 2, stage III = 3, stage IV = 4. BCLC stage: stage A = 1, stage B = 2, stage C = 3. Immune score: CD3_CT/IM_ CD8_CT/IM_ (CT, center of tumor; IM, invasive margin), minimum *P*value cutoff with two groups: Im2, 3, 4 = 1, Im0, 1 = 2. T stage (histopathologic criteria of tumor invasion): T1 = 1, T2 = 2, T3 = 3, T4 = 4. N stage (spread to local lymph nodes): N0 = 0, N1 = 1. Tumour thrombus: N = 0, Y = 1. CD3_IM_: low = 1, high = 2. CD8_IM_: low = 1, high = 2. CD3_CT_: low = 1, high = 2. CD8_CT_: low = 1, high = 2.

Abbreviations: DFS, disease-free survival; OS, overall survival; HR, hazard ration; CI, confidence interval.

* Log-rank *P* value corrected

a Multivariate analysis among UICC (TNM) stage, BCLC stage and immune score uses strata cox model.

† Significant. HR corrected

We further observed that CD8_CT_ performance on the disease parameters. It was noted that high densities of CD8_CT_ were highly significantly associated with high levels of alanine aminotransferase (ALT, *P* = 0.0004) (Figure 6C), aspartate aminotransferase (AST, *P* = 0.0002) (Figure 6D) and massive tumour volume (*P* < 0.0001) (Figure 6E), suggesting that CD8_CT_ cell densities also associated with the clinical characteristics of patients. However, no significant difference have been observed in the levels of alpha-fetoprotein (AFP, *P* = 0.2095) (Figure 6F). We also compared the relationship of IS to clinical characteristics of the HCC patients. The patients with higher IS exhibited a lower rate of recurrence or thrombus, but no correlations of tumour metastasis were observed to IS (Figure S2A). Interestingly, IS scores were also correlated with most clinical parameters, including AST (Figure S2C), AFP (Figure S2D) and tumour volume (Figure S2E), but no significant correlation between the IS and ALT (Figure S2B) or pathological grade (Figure S2F) have been observed. Our results suggest that CD8_CT_ densities analysis notably improved the accuracy of survival prediction if compared with IS, UICC-TNM and BCLC stages. As a result, taking account of convenience of clinical manipulation, we recommend the direct use of CD8_CT_ as an indicator for prognosis. The minimum *P* value cut-offs value 93 cells/mm^2^ can be used to divide the patients into CD8_CT_^Hi^ group and CD8_CT_^Lo^ group in clinical settings.

## DISCUSSION

HCC is a major public health problem with an annual incidence of more than 700,000 cases in the world. Simple and effective prognostic markers are needed to predict the survival, which is helpful to avoid under/over treatment in HCC patients in clinic settings. Herein, we evaluated the densities of CD8^+^ cells and CD3^+^ cells in PT and IT regions in a small Cohort 1 (90 patients), and observed a positive correlation between densities of lymphocytes and the overall survival time of patients was existed in CT region, but not in PT region. Based on this, we delivered to use a big Cohort 2 (359 patients contain the IM and IT regions) to investigate the prognostic capability of the infiltrated lymphocytes. We observed that patients with higher densities of CD3^+^or CD8^+^T cells in both regions exhibited significantly longer DFS and OS (Figure 4A, 4B). In order to more efficiently predict the survival, we combined the indicators of CD3^+^or CD8^+^T cells in CT or IM regions into a simple scoring method (IS), as established in colon cancer and rectal cancer [4, 6, 8-10], in our Cohort2. Our results demonstrated that patients with lower IS was significantly associated with poor prognosis, and patients with same TNM stage could even be clearly divided into two distinct groups, suggesting IS has an advantage over or compensates for current TNM tumour classification. However, using multivariate analysis model, we found the prognostic performance of the combinational IS (e.g. combining the density of CD8_CT_, CD8_IM_, CD3_CT_, CD3_IM_) was not advantaged over single analysis of the density of CD8_CT_. Patients with higher density of CD8_CT_ cells not only strongest predicted the HCC survival but also associated with significantly high levels of ALT, AST and massive tumour volume (Figure 6C-6E). Importantly, we found that the density cut-off of CD8_CT_ cells (e.g. 93 cells/mm^2^) can be used in future clinical settings to divide the CD8_CT_^Hi^ group and CD8_CT_^Lo^ group (Figure 4 and Figure S3C). Taken together, our data indicates that the density of CD8_CT_ cells is a better indicator to predict the prognosis of HCC.

The predictive accuracy of IS staging system was first demonstrated in colorectal cancer (CRC) patients in 2006 [12]. Accumulating clinical data have suggested that the prognostic value of IS classification in CRC patients is superior to the AJCC/UICC TNM-classification [4-5, 7-8, 12-13]. Recently, the predictive value of IS system was reported in rectal cancer, melanoma and breast cancers patients [10]. To our knowledge, the predictive role of IS system in HCC has never been reported. As reported by us, the HCC patients with higher ISs are significantly associated with longer DFS and OS compared with lower ISs (Figure 5A, 5C). Univariate Analysis also confirmed the differences between IS and survival in HCC (HR of 1.63 for the DFS and HR of 1.69 for OS, both *P* < 0.0001; Table 1) is even more significant than that in rectal cancer patients (HR = 1.81, *P* = 0.0038 for the DFS and HR = 1.72, *P* = 0.0003 for OS).

Tumour-infiltrating CD8^+^ T cells have been associated with favourable outcomes for patients with multiple tumour types. In patients with similar-staged urothelial carcinoma, significantly longer DFS and OS have been observed in patients with a higher numbers of CD8^+^ cells in the CT regions by Kaplan-Meier curve [14]. Multivariate survival analysis revealed that tumour infiltrating CD4^+^ T^high^/CD8^+^ T^high^/%Treg^low^ significantly correlated with longer survival time in 212 pancreatic cancer samples [15]. Analogously, this prognostic effect of CD8_CT_ constantly been confirmed in a variety of tumours such as epithelial ovarian cancer [16-17], breast cancer [18], prostate cancer [19], renal cell cancer [20], non-small cell lung cancer [21], colorectal cancer [22] and esophageal cancer [23], suggesting the great potential of tumour-infiltrating CD8^+^ T cells as a predictor for survival. However, with relatively few reports, the prognostic performance of CD8^+^ cells is still controversial in HCC. On the one hand, two laboratories have confirmed that CD8^+^ T lymphocytes in CT regions strongly correlated to longer DFS in HCC [24-25]; on the other hand, interestingly, one of the same laboratory has reported a opposite conclusion in another study [26]. Possible causes for this contradiction are the selection of cut-off value and patient number in cohorts. Both articles used median values as cut-off (median: 35 and 17.74 per HPF, respectively) in two cohorts with patient number 123 and 302, respectively. In our study, based on comparison among several analysis methods and utilization of big cohort, we conclude that the density of CD8_CT_ cells is the most valuable indicator to predict the survival of HCC, with the 93 cells/mm^2^ as the cut-off value.

It has been observed that tumour infiltrating lymphocytes can influence the outcome of patients with certain tumour types [27]. Recent advances demonstrated that a variety of immune cells such as CD4^+^ (T helper cell), CD45RO^+^ (memory T cells), CD56^+^ (NK cells), Foxp3^+^ (regulatory T cells) and IL-33^+^ (interleukin-33-producing cells) cells were associated with prolonged survival in liver tumours [28-30], however, comparing which, CD3^+^ and CD8^+^ cells were considered as the most promising candidates [5, 7-8, 12, 26]. In this study, the Cox multivariate model showed that CD8_CT_ cell is an independent parameter to better predict the DFS and the OS than other immune-related indicators. Based on our results from Kaplan-Meier curves and Cox multivariate model, the single analysis of central tumour CD8^+^ density is proved to be sufficient to achieve the desired prognostic power, making this immune prediction become easier, reproducible and even more economic in future practice.

According to univariate analysis, as shown in Table 1, tumour number (HR: 2.28, *P* < 0.0001), metastasis (HR: 1.62, *P* = 0.0264), tumour thrombus (HR: 2.94, *P* < 0.0001) and TNM stage (HR: 1.28, *P* < 0.0001) were significant prognostic indicators for DFS in the patients. However, a gradual HR decrease of DFS was not observed along with the raise of TNM stages. The BCLC staging, AFP, HBV infection, HCV infection and pathology grading have no statistical prognostic impact on DFS and OS of patients. Regarding tumour recurrence, we found the densities of CD3^+^ and CD8^+^ cells were significantly higher in both CT and IM regions in patients without tumour recurrence (Figure 3A). Inversely, patients with tumour relapse predominantly had a low densities of CD3^+^ and CD8^+^ cells in both regions of the primary tumour. Consistent with studies of Galon et al, a gradual decrease of the percentage of relapse patients were observed along the increase of ISs (Figure S2A). These data strongly suggests that local immune capacity in tumour regions is critical to prevent tumour recurrence. In addition, although a positive correlation of the tumour size with UICC-TNM stages were observed, the CD8_CT_ number alone was not correlated with TNM stages (Figure 4D), suggesting that tumour classification based on histopathological parameters were lack of providing information about the immune reaction in tumours regions and consequently poor to predicting survival time and tumour recurrence.

Even though the central CD8^+^ density seems to be biologically meaningful and clinically reproducible, some issues should be considered before a routine clinical examination is initiated. In Cohort 1, we have observed the overall survival difference between patients with different densities of CD8_CT_ (Figure 1). Such differences became even more significantly with the increased number of samples in Cohort 2 (Table S2), indicating a more accurate and reliable predication performance should be determined by a large number of patients. We have counted the positive points of CD8^+^/CD3^+^ cells in 50 random patients by pathologist and software respectively. Both calculation results are consistent in linear regression models (for CD3^+^ cells, R = 0.952; for CD8^+^ cells, R = 0.978; P < 0.0001 for both). These results verified the availability of software automate measurement(Figure S3). In our results, the overall survival time indeed decreased at 50-60 mouths post surgery. An immune therapeutic boosting for the patients with higher IS such as cytokine-induced killer cell, dendritic cell, natural killer cell therapy and other treatment of active potential T cell responses would be considerable to improve their survival time [31]. TMAs in our study are mainly focus on revealing prognostic impact in large populations. Since the importance of sensitivity for every patient in clinical practice, whole slides of tumour regions should be used which can improve the accuracy of tumour localization and density calculation. The digital pathology and image analysis software may also improve the accuracy and comparability.

In summary, our findings confirm and extend to patients with HCC the density of CD8 cells in CT regions as a valuable marker in predicting patient survival time andtumour recurrence. Specifically, patients with the same TNM or BLCL stages could be clearly divided into two groups using the CD8_CT_ density “93 cells/mm^2^” as a cut-off value. In each stage, patients with CD8_CT_ densities > 93 cells/mm^2^ have significantly longer survival compared with patients with CD8_CT_ densities < 93 cells/mm^2^. We further determined that the CD8_CT_ density is a prognostic factor superior to IS classification in the Cox multivariate analysis model. Simple analysis of the density of CD8_CT_ is already met the needs for survival prediction, which is economical and easy to be reproduced in clinical routines. For the first time, our results illustrate the significance of the CD8_CT_ densities as an independent prognostic index in evaluating the prognosis in HCC patients.

## MATERIALS AND METHODS

### Patients and classification

This study involved two independent cohorts of HCC patients (Table S1). Paraffin-embedded tumour samples were prospectively obtained from 90 patients who underwent curative resection between 2006 and 2009 (designated Cohort 1). The TMAs contained 90 patients diagnosed with HCC in Taizhou Hospital of Zhejiang Province were purchased from Shanghai Outdo Biotech Company. Tumour samples from 359 untreated patients with pathologically confirmed HCC and underwent curative resection between 2002 and 2003 were obtained from the Bank of Tumour Resource at the Sun Yat-Sen University (designated Cohort 2). The main difference between cohort 1 and cohort 2 is the absence/presence of invasive margin (IM). The tissue samples from cohort 1 possess both PT and IT regions, while those from cohort 2 possess PT, IT and IM regions. The clinical characteristics of all patients are summarized in Table S1. The details of all patients have been provided according to REMARK in Table S3 [32]. All tumour samples were anonymously coded in accordance with the Declaration of Helsinki. The protocol of all study cohorts were approved by the Ethical Board of the Institutional Review Board of the University of Science and Technology of China.

### Immunohistochemistry

The paraffin sections were dewaxed in xylene and rehydrated with distilled water. Following incubation with antibodies against either human CD3 (LN10) (Leica Biosystems) or human CD8 (SP16) (Biocare), the adjacent sections were stained with DAB Peroxidase Substrate Kit (SK-4100) and VECTOR VIP Peroxidase Substrate Kit (SK-4600) (Vector Laboratories). The positive cells were quantified using ImagePro Plus software (Media Cybernetics). All section stained TMA slides were examined by the reviewers who have no knowledge of any clinical data. Two cores of CT and IM regions were taken from the primary tumour as previously described.

### Immunoscore calculation

Precise quantification was performed under the combination of two markers (CD3^+^ and CD8^+^) in two regions (CT and IM). High density of each marker in each region recorded as a score and low density without scoring. For instance, Immunoscore 0 (I0) was low densities of both cell types are found in both regions and Immunoscore 4 (I4) was high densities are found in both regions [4-5, 8, 13].

### Statistical analysis

Statistical Product and Service Solutions (SPSS) statistics software (version 22.0) was used for all statistical analyses. Kaplan-Meier estimates of survival were used to illustrate the survival curves and to obtain the estimators of the median and survival rates for OS and DFS. The correlation between the densities of immune cells and survival time were using Cox regression with time-to-event outcome analysis. Significant differences between groups were determined using unpaired two-tailed t tests unless otherwise specified; P < 0.05 was considered significantly different.

## SUPPLEMENTARY MATERIAL FIGURES AND TABLES




