# Supplementary material for: The predictive value of centre tumour CD8+ T cells in patients with hepatocellular carcinoma: comparison with Immunoscore
Source: Oncotarget. 2015 Sep 22;6(34):35602–15. doi: 10.18632/oncotarget.5801 (PMC4742128; doi:10.18632/oncotarget.5801)
Supplement: Supplementary file 1 [file oncotarget-06-35602-s001.pdf]

The predictive value of centre tumour CD8^{+} T cells in patients with hepatocellular carcinoma: comparison with Immunoscore

Supplementary Material

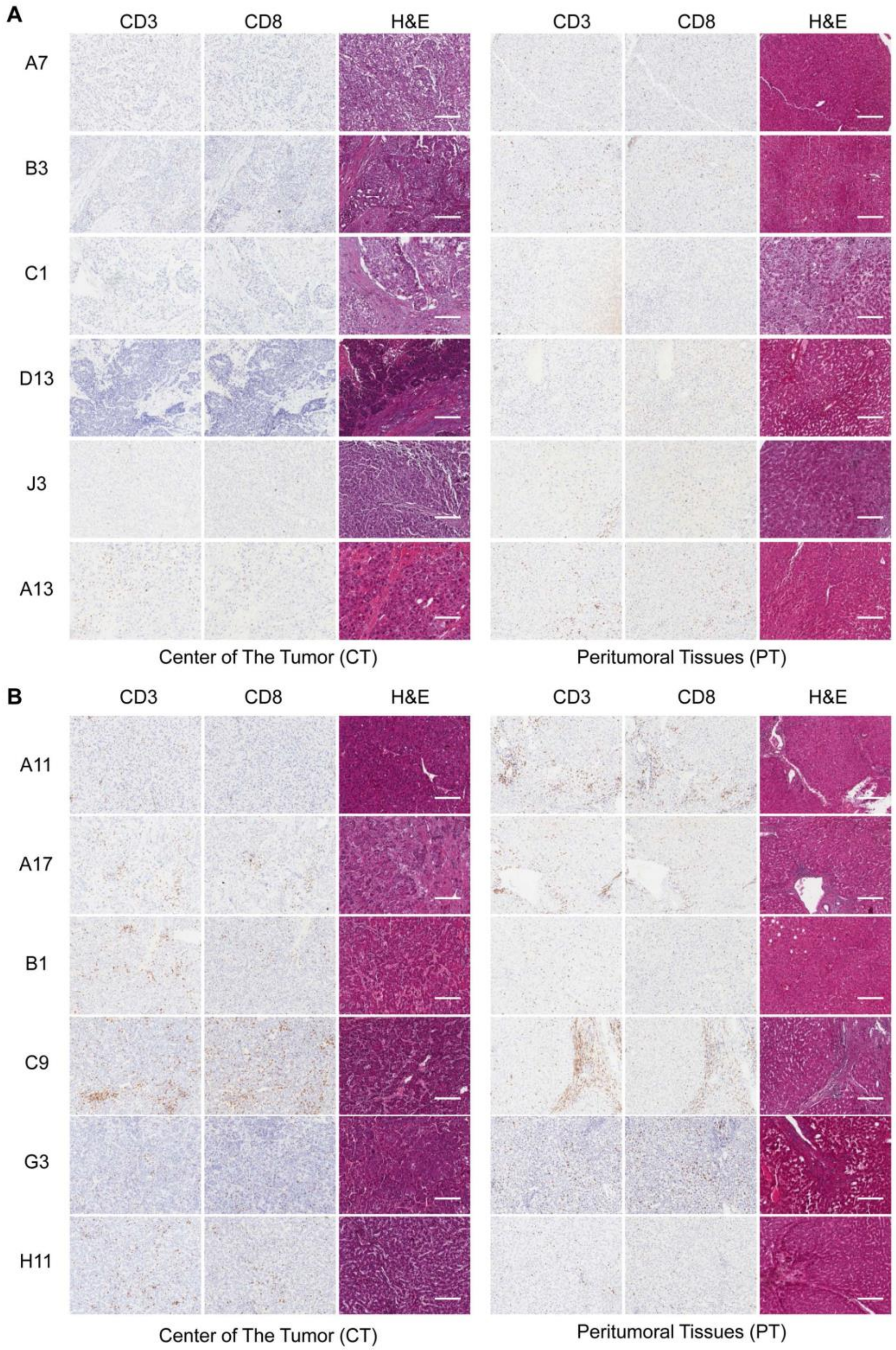

Figure S1. Immunohistochemistry of tumour-infiltrating CD3+ or CD8+ cells in 6 HCC patients. Adjacent sections of paraffin-embedded hepatoma samples stained with anti-CD3 or anti-CD8 antibodies. (A) Low levels of CD3+ or CD8+ cells can be visualised in the CT and PT regions. Original magnification: ×10. Bar=200 μ m. (B) High levels of CD3+ or CD8+ cells can be visualised in the CT and PT regions. Original magnification: ×10. Bar=200 μ m.

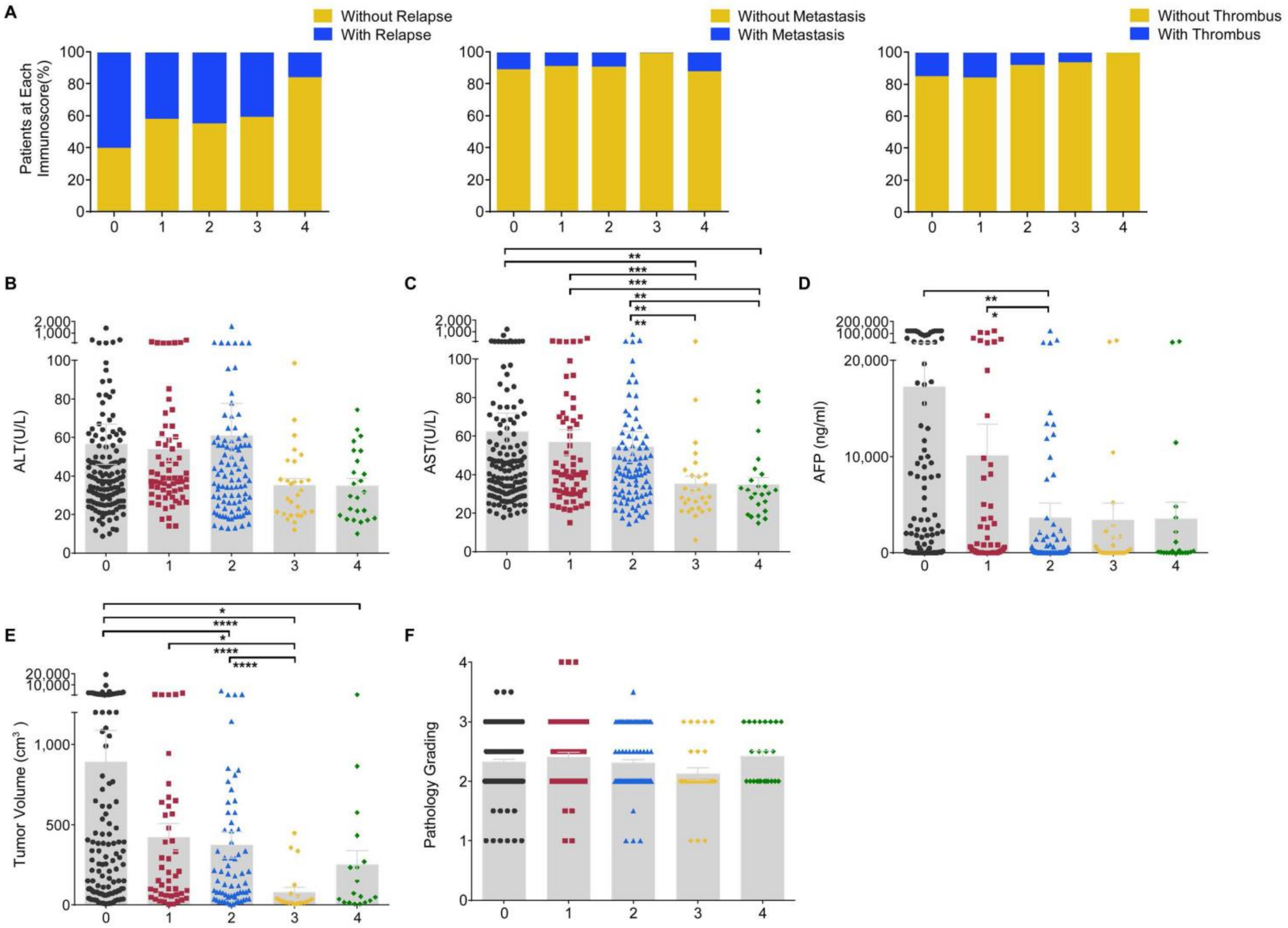

Figure S2. Clinical characteristics of HCC patients with different IS.

(A) The percentage of patients in each IS with or without recurrence, metastasis or thrombus. ALT (B), AST (C), AFP (D),Tumor volume (E) and Edmondson-Steiner (F) for all HCC patients with IS 0 (Black), IS 1 (Red), IS 2 (Blue), IS 3 (Yellow) or IS 4 (Green). *, $P < 0.05$; **, $P < 0.01$; ***, $P < 0.001$, ****, $P < 0.0001$.

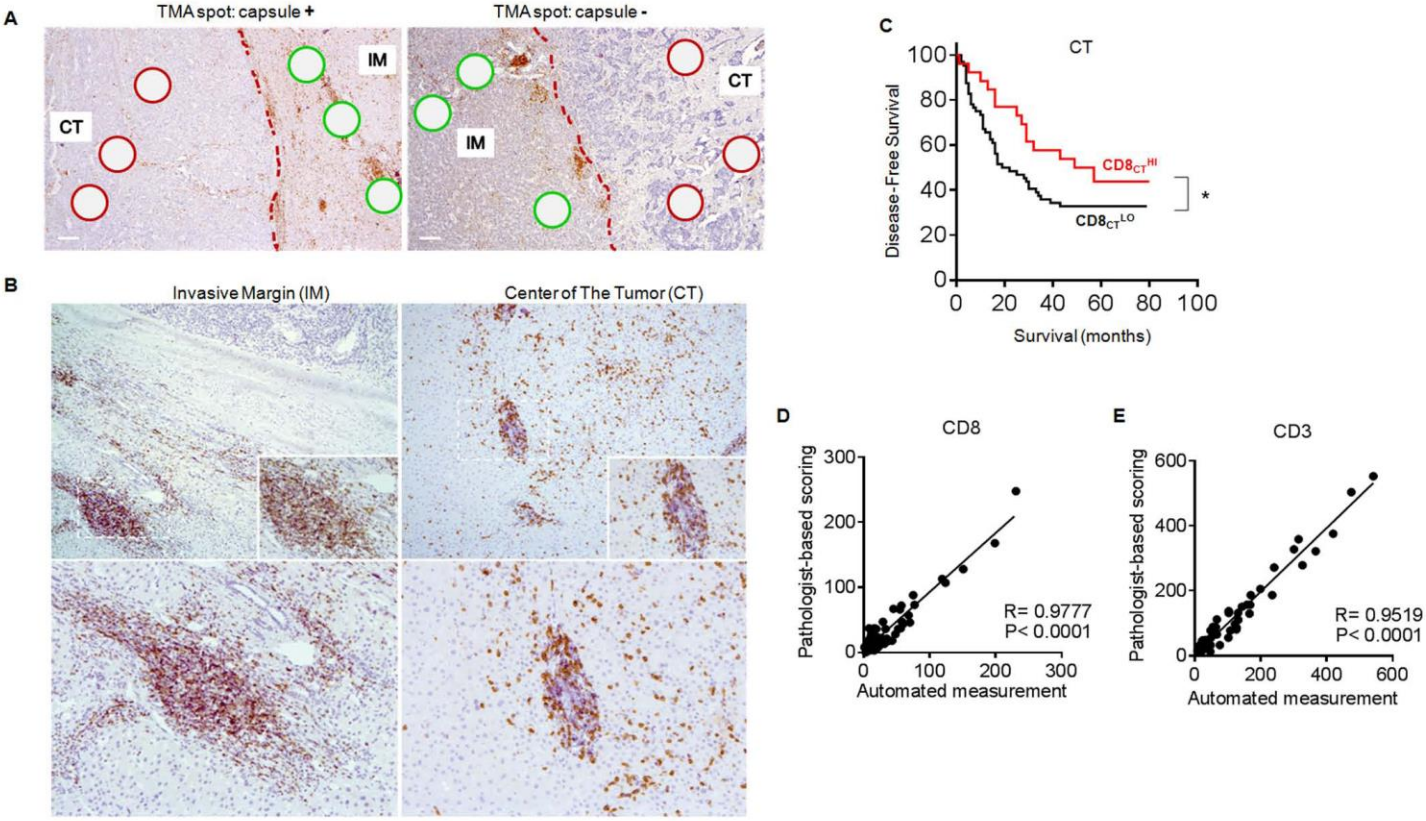

Figure S3.(A)The select strategy of the CT(red) and IM (green) regions intissue microarrays (TMAs). The dashed lines represent the edges of the tumour or the IM. Original magnification: ×5. (B)Representative micrographs showing double staining of CD3+ (Brown) and CD8+ (Purple) cells in the IM region (Left) and CT region (Right) with an original magnifications of 10× (Top) and 40× (Bottom). (C) Kaplan–Meier curves for the duration in months of OS according to CD8+ cells densities evaluated in CT regions from patients of cohort 1. (D, E) The correlation between pathologist-based scoring and software automated counting of the positive points of CD8+/CD3+ cells onimmunohistochemical photos from 50 random HCC patients.

S.Table 1. Clinicopathological characteristics in each cohort and in the total material

Group	Cohort 1	Cohort 2
Number of patients	90	359
Sex (male)	78 (86.7%)	318 (88.6%)
Age (years)	53.5 ± 10.0	50.0 ± 13.7
Time of inclusion	2007-2009	2002-2010
Last follow-up	Sep 2013	Jul 2014
Endpoints	OS	DFS, OS
OS(days)	33.2 ± 24.0	1299.7 ±1974.2
DFS(days)	----	980.0 ±1796.1
Tumor Volume(cm^3)	406.3 ± 661.1	577.8 ±3.9
Metastasis negative	84 (93.3%)	293 (81.6%)
Cirrhosis positive	33 (36.7%)	178 (49.6%)
ALT (U/L)	----	54.0 ±220.6
AST (U/L)	----	55.1 ±438.4
ALB (g/L)	----	42.6 ± 1.3
AFP (ng/ml)	----	10306.4 ± 4021.4
TBIL (μmol/L)	----	16.5 ± 0.9
Recurrence	----	175 (48.7%)
Tumour thrombus	----	43 (12.0%)
Capsule	----	257 (71.6%)

Abbreviations: DFS, disease-free survival; OS, overall survival; IHC, immunohistochemistry

S.Table 2. Univariate Analysis of DFS and OS Among Patients With Liver Cancer According to Immune Parameters

	DFS			OS		
Parameter	HR	95% CI	*P* *	HR	95% CI	*P* *
**Corhort 1**						
$CD3_{CT}$				0.999	0.998 to 1.000	0.1082
$CD8_{CT}$				0.998	0.995 to 1.001	0.1231
$CD3_{PT}$				1.001	1.000 to 1.002	0.0351
$CD8_{PT}$				1.001	0.999 to 1.003	0.2625
**Corhort 2**						
$CD3_{IM}$	0.998	0.997 to 0.999	<0.0001†	0.997	0.996 to 0.999	<0.0001†
$CD8_{IM}$	0.996	0.995 to 0.998	<0.0001†	0.996	0.994 to 0.998	<0.0001†
$CD3_{CT}$	0.996	0.994 to 0.997	<0.0001†	0.995	0.993 to 0.997	<0.0001†
$CD8_{CT}$	0.992	0.989 to 0.995	<0.0001†	0.99	0.987 to 0.994	<0.0001†

Abbreviations: DFS, disease-free survival; OS, overall survival; HR, hazard ration; CI, confidence interval; CT, centre tumour region; PT, peritumour region;

†Significant.

Table 3: Clinicopathological characteristics of HCC patients
